# Patients’ mobility as an indicator for (in)efficiency:a panel data analysis on Italian health care authorities

**DOI:** 10.1186/2191-1991-3-3

**Published:** 2013-02-19

**Authors:** Elisabetta Mafrolla, Eugenio D'Amico

**Affiliations:** 1 Department of Economics, University of Foggia, via Caggese 1, Foggia 71121, Italy; 2 Department of Public Affairs, Rome Third University, Rome, Italy

**Keywords:** Health care management, Efficiency in health care, Health treatment mobility, Operating costs in health care, Fixed effects panel model, Italian health care authorities

## Abstract

**Abstract:**

This paper investigates the influence of internal managerial patterns of heath care authorities on the decision of patients to migrate towards different health care organizations to avail treatments. The efficiency and productivity issues are analyzed, considering the (passive) migration as a proxy for the (in)efficient service availed. We follow the “vote by feet” theorization by Tiebout , assuming that citizens can choose to avail a health treatment in a public service provider different from their resident one. The choice for a center that is far from home implies a negative judgment to the alternative health care supplier that is closer to the patient. Testing Fixed Effects Panel Model on a sample of Italian health care authorities, a strong correlation is found among variables in our model and some relevant dependence is tested between patients’ mobility behavior and their resident authorities’ efficiency in allocating resources on the proper operating cost. Spending in the proper way on health care could bring about an enhancement of performances. Instead, wasting resources is immediately perceived by the patient, who consequently seems to move to a different health care authority.

**JEL code:**

M48

## Background

Various empirical studies, investigating efficiency in health care [1], distinguish themselves primarily by the object of the investigation, then by the model adopted, and finally by the parameters chosen.

The larger target of essays in the available literature is the hospital, in its various configurations [1-3], searching similarities and differences between the relevant characteristics of the clinic, like public and private proprietorship and management [4,5], for-profit and not-for-profit organizations [6], specialist and general hospitals [7]. Another important branch of studies deals with national and local health care programs and systems [8]. Much more limited is the number of works about the health care peripheral authorities (or districts), which can be investigated on the micro, macro, and meso levels.

The second important question is the choice of the research empirical model adopted. We basically distinguish three main categories of studies. Farrell [9] proposes the fortunate data envelopment analysis (DEA), a parametric test to individuate which entity is closer to an optimal ideal frontier. The stochastic frontier analysis (SFA) [10] is the most important alternative parametric method adopted to individuate the productivity of organizations. Lastly, the classic value based analysis (VBA) unleashes the existing debate on the choice between parametric and nonparametric methods [11]. Being generally consistent with both the other studies, it offers a less mathematical and statistical sophisticated model, looking for a simple ratio analysis and a linear or nonlinear welfare function in order to investigate the efficiency issue [12,13].

In order to measure efficiency correctly, several different production functions are proposed, which barely assimilate the productivity angle into the efficiency issue, focusing instead on questions related to technical and technological efficiency [14,15]. Researchers propose various forms for that function, some of which had great academic approval [16,17], others less [18]. In any case, a common set of rules for the proper measurement of efficiency in health care is still lacking. The concept of efficiency itself is variously used by researchers with different scopes, causing subsequent difficulties in the comparison and generalization of findings [19].

Overwhelmingly, literature follows the “new public management” approach [20], looking for a precise and objective measurement of the efficiency, thus studying the issue largely from the clinical perspective, often excluding the patient’s perception of service quality [21]. Nevertheless, in the last 30 years, an interesting branch of studies analyzes the quality of health care services [22], even investigating the satisfaction of the patients. With that aim, many measures of patients’ satisfaction have been developed, making it difficult to individuate the most sensible and widely applicable one [23]. Furthermore, comparing the results across different researches is quite difficult due to the polymorphic structure of the conducted surveys and due to the emotional and subjective involvement of the patients interviewed [24].

This paper investigates whether and how in public health care (in)efficient spending decisions influence positively (negatively) the preference of patients for a medical center rather than others.

Our research is located in the field of empirical research on health care economics, investigating the question of the (in)efficiency in health care authorities (HCAs), adopting a VBA approach. We particularly appreciate the VBA because it gives the opportunity to monitor the main characteristics of the organization over space and time. After studying the behavior of the sample over the given period and noticing the effect of different national and regional policies on our indicators, we use the econometric panel modeling to regress data and infer relations between the various characteristics of the HCA as a firm.

Furthermore, we locate our research in the field of regional studies, choosing a sample of HCAs operating in Apulia, Italy, over the period 2001–2010. To our knowledge, a few academic analyses empirically investigate the efficiency issue in health care in Italy and most of them are focused on cases from northern Italy [25-29]. Only Dell’Anno & Longo [30] inspect a case from Southern Italy, focusing on hospitals and HCAs in Apulia, adopting the SFA and finding interesting positive relations between the cost efficiency and the presence of university departments in the medical center.

Compared to available Italian literature, our study involves a case from southern Italy, investigates a longer period of time (nine years, instead of the four analyzed by Dell’Anno & Longo), and adopts different variables and research model (as described below), considering the HCA as a firm, with the aim of maximizing the results and optimizing the expenses.

The paper is structured as follows: after a brief introduction to the issue and to previous literature (part I), in part II, we describe our research question and locate it theoretically; in part III, we describe methods and sample and give a snapshot of HCAs in Italy, and particularly, a brief overview of the last 10 years of public health care in Apulia (our sample); in part IV, show results; and finally, in part V, we discuss some relevant findings.

### Theory and research development

We study the (in)efficiency issue in health care from an unusual perspective, the one of HCA as a firm, adopting an innovative way of measuring the performance. In our view, efficiency is a measure of the performance of the firm, calculated as the relation between the outcome (i.e., the migratory phenomenon) and the consumption of resources (i.e., our clinical and monetary independent variables) that characterize the analyzed production process.

Our dependent variable measures the mobility of the population for health treatments from one HCA to another. We consider this variable as a good proxy for quality in health care services provided in a HCA due to the fact that mobility is basically driven by the expectation of the quality of services supplied in the HCA of destination, compared to a lower quality experienced (or expected) in the resident HCA. Thus, the patients value the relative quality of services provided in different HCAs more than the easier access to those supplied in the proximity of their own resident HCA.

The use of the mobility factor as a proxy for efficiency in public services is actually well documented in the economic literature. More than 50 years ago, while explaining the basic difference between the provision of national and local services, Tiebout [31] stated that for those services supplied at the local level, “the consumer-voter moves to that community whose local government best satisfies his set of preferences.” (p. 418) In Italy, the national health care system provides a main common framework of service supply (and related payments), which can only partially vary at the local (i.e., regional) level. Thus, basically, most of the services are supplied all over the nation at the same cost to the patient. Furthermore, in Italy, the patients have a fundamental right to choose the medical center that they trust more. Thus, the theory by Tiebout could find an interesting empirical test in the Italian HCAs system, where the phenomenon of mobility can be observed.

Our first hypothesis is the existence of a relationship between the different inputs of health care supply and the judgment implicitly given by the patients migrating out in search of a higher quality of services. Inputs of health care supply, in our managerial perspective, are basically the various structures of operating costs adopted by each HCA (considered as a firm). With that aim, we try and investigate the different patient behaviors changing the typology of expense. Spending on some factors of production actually could be a waste. Spending on other constituents of the production process instead could be an investment. We hypothesize that the patients value the operating cost structure of the HCA and move from one to the other (i.e., migrate) by valuing the efficient spending choices.

Our second hypothesis tries to partially clear the biggest hurdle while dealing with Tiebout’s assumptions: the bias of the perfect mobility of patients. Dividing the total migration of patients into extra-regional and infra-regional phenomena, we test the relevance of the spatial pattern in the “vote by their feet” decision of the patients.

## Methods

### Sample description

The sample is a panel composed of nine time series (years 2001 to 2009) and six cross sections, representing the HCAs located in Apulia, totally 54 firm-year observations.

The HCA (*Azienda Sanitaria Locale* - *ASL*) is the public peripheral entity supplying health care services in Italy. HCAs work with internally managed clinical centers and provide basic medical services of prevention, diagnosis, and the treatment of diseases. Clinical centers in Italy can even be run in autonomy, out of the HCAs’ control, both in a public and private juridical and economic capacity.

The number and composition of the HCAs in Apulia notably changes over time (see Table 1). The Italian national health care system in 1992 (d.lgs. n. 502) devolved autonomous powers (and responsibilities) to local health authorities and asked for a rationalization of costs and efficiency of services. In the second half of the 1990s, the 55 existing HCAs were merged, creating 12 HCAs and four autonomous public hospitals (*Aziende Ospedaliere*). Many changes occurred in the past 15 years in the legal structure and the economic organization of the regional health care systems. In 2001, the national law gave further powers to the regional authorities, which carried on several spin-outs and mergers of medical centers. In 2005, the constitution of a new provincial administration caused the spin-off and merger of the sector, with the creation of the new Barletta-Andria-Trani HCA. On January 1, 2007, the important merger of three groups of HCAs defined a rigid division of the HCA per province (Bari, Brindisi, Barletta-Andria-Trani, Foggia, Lecce, and Taranto, in acronyms respectively BA, BR, BT, FG, LE, TA), ranking the BA HCA among the most populous health care districts in Italy. The 2011 landscape of Apulia regional health care system presents six HCAs; two autonomous public hospitals and two institutes for health care and research (so called *Istituto di Ricovero e Cura a Carattere Scientifico – IRCCS*) complete the public supply of health treatments in Apulia.

**Table 1:** Spinn-offs and mergers in Apulia health care system over last decade

**Cross section units**	**2001-2004**	**2005-2006**	**2007-2010**
**BA**	BA/1	BA/2 (plus part of ex-BA/1 and minus 3 towns)	BA (merger of BA/2, BA/3, BA/4, BA/5)
	BA/2	BA/3	
	BA/3	BA/4	
	BA/4	BA/5	
	BA/5		
**BR**	BR	BR	BR
**BT**	NOT EXISTING	BT (including most of ex-BA/1, 3 towns of BA/2 and 3 towns of FG/2, spinned-off and merged)	BT
**FG**	FG/1	FG/1	FG (merger of FG/1, FG/2, FG/3)
	FG/2	FG/2 (minus 3 towns)	
	FG/3	FG/3	
**LE**	LE/1	LE/1	LE
	LE/2	LE/2	
**TA**	TA	TA	TA

### Description of variables

We summed up information on activities, costs, and population, considering the mergers occurring over time as if they occurred in 2001.

We collected the dataset made up of different kinds of variables.

First, the regressand is a measure of the migration from the resident HCA towards other HCAs (out or in the regional limits). As explained earlier, we analyze the overall migration, decomposing it into the extra-regional migration scaled by population (*ERM*) and the infra-regional migration to population (*IRM*). *IRM* suffers less than *ERM* due to the bias of the spatial sacrifice to reach the selected service supplier. We calculate migration as the difference between the number of inhabitants of the target HCA who avail a health care service in a different HCA (passive migration) and the number of patients coming to the target HCA for treatments (active migration). This difference in Apulia is always a passive balance of migration; consequently, we omit the minus sign and always consider the prevalence of the passive over the active migration in our index.

Second, the regressors explain different characteristics:

i. The adequacy of infrastructures available in a HCA to the needs of the resident population: We calculate *OBTB* as ratio of the number of occupied beds in one year to the number of available beds. We add two dichotomic variables to isolate the cases of extreme under-utilization (*DummyUNDER =* 1 if *OBTB* < 0.65, 0 otherwise) and over-utilization (*DummyOVER =* 1 if *OBTB* > 0.89, 0 otherwise) of assets and general costs.

ii. The epidemiologic and demographic characteristics of the inhabitant population of the target HCA: We consider the hospitalization index *OBPOP*, calculated as ratio of the number of occupied beds to the resident population, a proxy for those characteristics.

iii. A series of typical managerial parameters, measuring the costs of the HCA as a firm: Thus, we reported:

- *MEDPERS* (= Income Statement item #B00800), costs of the medical personnel to the population;

- *OTHPERS* (= Income Statement items #B00810 + #B00820 + #B00830), costs of professional, technical, and administrative human resources to the population;

- *PHARM* (= Income Statement item #B01005), expenses for surgery and medical materials and pharmaceuticals to the population;

- *TOTPROD* (= Income Statement item #B99999), total typical costs of production (B area of the income statement) to the population.

The regressand and the variables, sub i) and sub ii), are deduced using data kindly provided by Svimservice Srl, a private company offering informative services to the Apulia Regional Authority. The variables in sub iii) are collected, elaborating the income statements publicly given out by the Italian Health Care Ministry. Listed variables are scaled by population, measured by the Italian Statistic Institute (ISTAT).

Some descriptive statistics are presented in Table 2.

**Table 2:** Descriptive statistics

	**Mean**	**Median**	**Min**	**Max**	**St.dev.**	**Var.coeff.**	**Skew.**	**Curt.**
***OBTB***	0.69708	0.74856	0.19017	0.95182	0.16169	0.23196	−1.4706	2.1637
***OBPOP***	0.92309	0.94209	0.53816	1.1612	0.15893	0.17217	−0.61933	−0.2627
***DummyOVER***	0.05556	0	0	1	0.16169	4.1618	3.8806	13.059
***DummyUNDER***	0.24074	0	0	1	0.15893	1.7926	1.2128	−0.52908
***MEDPERS***	293.07	292.96	174.97	431.67	76.284	0.26029	−0.0052274	−1.2854
***OTHPERS***	70.314	68.434	51.326	90.778	12.090	0.17188	0.14327	−1.3795
***PHARM***	139.97	122.76	56.962	282.87	55.42	0.39596	0.74423	−0.12039
***TOTPROD***	1448.30	1527.30	721.59	1791.60	266.63	0.18410	−1.3573	1.2621
***ERM***	0.011148	0.012417	0.0021194	0.018535	0.0040462	0.36295	−0.59507	−0.65582
***IRM***	0.030131	0.027478	0.0064730	0.066611	0.019265	0.63938	0.25064	−1.4679

About the dependent variables, at a glance, the migration indexes show that, on average, more than 4% of the resident population crosses the HCA’s borders to get health treatments and 1% goes even past the regional limits. The maximum value of the *IRM* (7%) and *ERM* (2%) indexes confirm the opinion that Apulia suffers one of the strongest health care passive migration phenomena in Italy. The distribution of *ERM* is not too far from the Gaussian, with *skew.* = −0.56 and *kurt.* = −0.65, while *IRM* is more symmetric (*skew.* = 0.25) and quite platikurtic (*kurt.* = −1.47). These conditions predict a good probability of encountering a normal distribution of the idiosyncratic errors after the regression analysis. In the end, the correlation between the two dependent variables is quite low (*ρ* = 0.21; *p-value* = 0.12). Thus, we expect different findings from the parallel analysis of the two components of the passive migration phenomenon.

The *OBTB* index has an average value of 0.69 and a standard deviation of 0.16. If the index were too close to 1, the motivation of migration could be strictly joined to the inadequate capacity of the services offered. If the index were too low, the infrastructures could be inefficiently oversized. Thus, considering that *max*(*OBTB*) = 0.95 and *min(OBTB)* = 0.19, we can state that the infrastructure is generally not adequate to the needs because of situations pertaining both to the deficiency and superfluity in investments. With the aim of measuring separately the issues of under-utilization and over-utilization, we add two dichotomic variables. *DummyUNDER* equals 1 when the *OBTB* gets to excessively low levels (*OBTB <* 0.65). *DummyOVER* equals 1 when the *OBTB* gets to excessively high levels (*OBTB >* 0.89). In the case of the physiological levels of *OBTB*, both *DummyUNDER* and *DummyOVER* equal 0. The distribution of the *OBTB* index is definitely asymmetric (*skew.* = −1.47) and sharply leptokurtic (*kurt.* = 2.16).

The hospitalization index (*OBPOP*) is generally quite close to 1, with an almost normal distribution (*skew.* = −0.61; *kurt.* = 0.26).

Average total operating costs per inhabitant (*TOTPROD*) are 1448 €; the minimum is 722 € and the maximum is 1792 €, with an asymmetric (*skew.* = −1.35) and leptokurtic (*kurt.* = 1.26) distribution. The distribution of other kinds of cost indexes (*MEDPERS*, *OTHPERS,* and *PHARM*) is quite symmetric (−1 < *skew.* < 1) and platikurtic (*kurt.* < 0).

Statistic significant correlations (Table 3) between independent variables are neither too high (*ρ* < 0.7) nor too low (*ρ >* 0.25), avoiding problems of perfect correlations and encouraging the probability of consistent findings from the regression analysis.

**Table 3:** Pearsons’ correlation analysis*

	***OBTB***	***DummyOVER***	***DummyUNDER***	***OBPOP***	***MEDPERS***	***OTHPERS***	***PHARM***	***TOTPROD***	***ERM***	***IRM***
***OBTB***	1.0000	0.363 (0.00)	−0.835 (0.00)	0.219 (0.11)	−0.294 (0.03)	−0.162 (0.46)	0.367 (0.00)	−0.065 (0.33)	0.093 (0.31)	0.171 (0.22)
***DummyOVER***		1.0000	−0.136 (0.00)	0.082 (0.55)	−0.062 (0.65)	−0.148 (0.28)	0.187 (0.18)	−0.264 (0.05)	−0.139 (0.31)	0.151 (0.27)
***DummyUNDER***			1.0000	−0.339 (0.02)	−0.3224 (0.02)	−0.159 (0.25)	−0.343 (0.01)	−0.156 (0.26)	−0.148 (0.28)	−0.054 (0.69)
***OBPOP***				1.0000	0.528 (0.00)	0.202 (0.11)	0.453 (0.00)	0.634 (0.00)	0.313 (0.02)	−0.171 (0.22)
***MEDPERS***					1.0000	0.681 (0.00)	0.675 (0.00)	0.604 (0.00)	0.390 (0.00)	0.233 (0.09)
***OTHPERS***						1.0000	0.376 (0.00)	0.517 (0.00)	0.123 (0.92)	−0.021 (0.14)
***PHARM***							1.0000	0.194 (0.32)	0.251 (0.07)	0.210 (0.11)
***TOTPROD***								1.0000	0.424 (0.00)	−0.169 (0.22)
***ERM***									1.0000	0.212 (0.12)
***IRM***										1.0000

* In brackets: two-tails *p-values*.

The variance inflation factors (Table 4) excludes the collinearity event for each independent variable included in our model (*VIFs* < 5.5).

**Table 4:** Variance inflation factors (VIFs)*

***OBTB***	5.368
***DummyOVER***	1.474
***DummyUNDER***	4.520
***OBPOP***	2.716
***MEDPERS***	4.383
***OTHPERS***	2.375
***PHARM***	2.606
***TOTPROD***	3.927

* Two-tails *p-values.*

### Data description

To measure the quality of health care supply in Apulia, we consider one of the most explicative trackers for a diagnostic test of (in)efficiency, studying migration flows between the HCAs over the decade 2001–2010. We use information on the clinical activity of HCAs kindly provided by Svimservice S.r.l. (under the authorization of the Apulia Regional Authority), who prepares the official regional database. We report the number of patients crossing the borders of the target HCA and availing health care treatments from other HCAs located in Apulia (infra-regional migration, in symbols: *IRM*) and out of the regional borderlines (extra-regional migration: *ERM*). These variables are scaled by population. As expected, most of the total migration is addressed to other HCAs of Apulia. To explain this phenomenon, we could enumerate many reasons out of our variables. First, the health care system is shaped on the idea of a capillary covering the whole regional territory (and population) only for basic treatments. Specialist clinics and hospitals are located only in a few strategic centers of excellence. Thus, to take advantage of a specialized medical center, the patient of a HCA has to often move to a neighboring HCA. Then, the *IRM* is consistent because it is planned *ex ante* in an overall regional health care system. Second, due to the geographic proximity of the other HCAs in the same region, patients who are not satisfied with the treatments they could receive by their HCA, can easily reach other HCAs within the region with a limited sacrifice of time and money. Lastly, only those who can financially afford the extra expense of traveling and lodging out of the regional borders decide to renounce the treatment in their own HCA, hoping to get services of higher quality in the elected HCA. Thus, we predict spatial proximity is a relevant bias in the “vote by their feet” decision of patients.

The trend in patients’ mobility slightly grows over the decade. It is easy to identify two different attitudes grouping the HCAs. The HCAs with a smaller number of inhabitants (BT, BR, TA) experienced a higher level of migration (around 0.6), that goes up over time. In the smaller HCA, BT, the internal mobility rapidly arises in 2005 and 2006, when the BT HCA is created. A different position is occupied by BA, FG, and LE, where the trend is either quite constant or decreasing (LE) over time and the *IRM* index assesses around 0.2. In those areas, important autonomous public hospitals mitigate the migration effect and attract people from other HCAs. We measure migration as the number of people crossing geographic borders, and the support for the presence of autonomous public hospitals is quite strong and lowers the intercept of the time series. The bias affects the infra-regional migration index, but not the extra-regional one, clearly reporting the choice of patients for a higher quality health care supply. People from BT and FG recurred to extra-region migration more than other HCAs patients.

The main structural independent variable we analyze is the number of beds provided in public hospitals and other clinics internally managed by HCAs^a^[32]. The total number of beds available in the whole region slightly deflects over the decade, from 15,440 beds in 2001 to 14,239 in 2010, losing 7.8% in 10 years, mirroring the national and regional policies of rationalization of offerings and reduction of costs. In 2001, 33% of the beds were in the most populous HCA (BA) and 25% were in LE (a much less populous area). The distribution keeps quite constant up to 2010, when 29% of the total beds are recorded in BA, 21% are in LE, 16% in TA, 15% in FG, 11% in BR, and 7% in BT. This unequal distribution is sensibly justified by the different concentrations of inhabitants. The relative configuration of the item (scaled by the number of inhabitants) is quite homogeneous among the different HCAs. On average, the total number of beds in hospitals and clinics (day hospitals included) represents 0.34% of the resident population. The ratio grows to a maximum of 0.49% (LE, 2001) and slopes down to 0.2% in BT. Most of the beds (12,887 in 2010) is dedicated to general hospitalization. Bed units for day hospital were totally 65 in 2001 and 816 in 2010. Wards assigned to rehabilitation opened in 2003, and in 2010, we count 536 beds for rehabilitation in Apulia HCAs’ internal hospitals. This finding is consistent with the decision of the regional government of changing some characteristics of the internally managed hospitals, partially converted, in those days, from general hospitals to rehabilitation clinics and specialized medical centers.

The adequacy of beds complementing the needs of the resident population is better investigated through the analysis of the intensiveness of the usage of structures, combining the number of occupied beds to the total bed units (*OBTB*). The ratio changes over time, registering a great increase in 2002 and 2003, lowering in 2004, and keeping a quite constant average rate up to 2010. The Italian national average of *OBTB* is 0.77. Levels of the index lower than 0.77 are registered quite often in Apulia in most of the HCAs, where the mean of the observations over the decade is 0.69. In order to isolate those cases where the index has underperformed, and where we expect inefficiencies due to excessive expenditure on overheads and general operating costs, we add to our model a dummy variable (*DummyUNDER*), counting 1 if the index goes below a physiological average level of utilization, and 0 otherwise. We measure that grey area as the average of the index minus the variance of index, computed at a national Italian level: *OBTB*_*i*_ < *Av*(*OBTB*_*It*_)-*var*(*OBTB*_*It*_) = 0.77-0.12 = 0.65. Contrarily, sometimes the index offers an image of overcrowded hospitals and clinics. In 7% of cases, it overwhelms the upper limit of 0.89, where *OBTB*_*i*_ > *Av*(*OBTB*_*It*_) + *var*(*OBTB*_*It*_) = 0.77 + 0.12 = 0.89. In those cases, we expect a high grade of scale efficiency due to the intensive utilization of assets and services, but a low satisfaction degree of patients, who probably suffer a low quality of health care supply due to long queuing and waiting lists. We try and extract the meaning of the outbalance of the described consequences of overcrowded medical centers, adding to the model a dummy variable (*DummyOVER*) that is 1 if the index goes above the physiological average level of utilization, and 0 otherwise. The above-limit is the described 0.89. We expect out-migration to be influenced in two opposite directions by the two exposed aspects of overcrowding in the HCAs’ medical centers. Thus, the predicted sign of *DummyOVER* is in doubt and the application to our case can help to understand which circumstance is more relevant to the patient. Actually, there is even an endogeneity problem in *OBTB*: if the medical center is overcrowded, it probably has a great appeal to the patients; on the contrary, a deserted health care department can make the patient suspicious about the quality of supply. Thus, the *OBTB* apparently influences a portion of the out-migration decision. We cannot adopt the GMM model to correct endogeneity due to N < 10. Thus, we appoint the national level of *OBTB* as an instrumental variable to our model and extract its significance, calculating *DummyUNDER* and *DummyOVER*.

The hospitalization index, calculated as the quotient *OBPOP*, measures the degree of utilization of the bed units, scaled by the number of resident population. With that aim, we capture the different dispositions of the population to availing care treatments, depending mostly on the demographic and epidemiologic factors. As expected, the variance in the index is not relevant and is more marked among HCAs than over the years. This is hardly a control variable to our model and we have no prediction as to the sign of the coefficient.

To explore specific reasons of (in)efficiency of the HCAs, we use Annual Financial Reporting and analyze how they spend money to supply services [33]. We appoint the most relevant items included in the income statement of the HCAs (data available for years 2001 to 2009), both for the magnitude and for the strategic relevance of the cost in a health care organization. To compare the information and find out resemblances and differences among the groups and over time, we divide the data by the resident population.

We study the cost of personnel, splitting it into physician (item #B00800 of the Income Statement) and non-physician (items #B00810 + #B00820 + #B00830) human resources (*MEDPERS* and *OTHPERS,* respectively). We find a regular growing trend of medical labor costs and realize that the difference existing between various HCAs in 2001 is kept over time and gets a little wider in 2009. On average, every year, each HCA spends 293€ per citizen to pay the salaries of physicians providing necessary public health care services. The increase in *MEDPERS* cannot be considered an evidence of inefficiency. Most of the academic empirical researches on patients’ satisfaction find a positive relation between resources invested in physicians and the patients’ perception of the quality of the health treatment [14]. Enhancing the expenditure, HCAs accumulate the number and quality of doctors offering treatments, diminishing the probability of patients’ migration. In 2010, 3879 family doctors (general and pediatric) were tenured in HCAs in Apulia, equally distributed on the regional territory in proportion with the number of inhabitants. In the same year, emergency clinical service was evenly well distributed among HCAs and occupied 1773 doctors, while 26% of outpatient physician personnel (totally 1734) worked in BA and 26% worked in FG, suggesting the possibility of an oversized allocation of resources in FG, where the population is smaller.

Even the cost of professional, technical, and administrative human resources (*OTHPERS*) slightly grows over the decade. Only in TA, a decrease of the cost is seen in four observed years (2003–2007), growing again in 2008 and 2009. Differences among the HCAs are quite marked, but the growing trend is almost contained everywhere since 2003. Probably the phenomenon is linked to the rationalization and merger strategies adopted first in 2002 and again in 2007, with the aim of cutting superfluous directional, administrative, and technical costs. Another tracker for (in)efficiency in the resource allocation is the total number of personnel tenured in a managerial position (both physician and non-physician employees)—6965 over a total number of 31,484 workers in the HCAs of the region. Directional and non-clinical costs’ intensification over time should be avoided in order to offer an efficient health care system. We predict the bigger the *OTHPERS*, the larger the inefficiency (and out-migration).

Another relevant cost category in health care is due to surgery, clinical and medical provisions, and pharmaceuticals (*PHARM* = item #B01005). Sensibly, this cost varies quite symmetrically over cross-sections and regularly changes its pendency over time, first growing, then lowering, and finally growing again. The regular path is due to some important biasing elements. First, the index depends on the general level of health of the population, which does not vary over time and space in a big way. Second, it is strictly correlated with regional reimbursement policies. Then, changing the policy, even the pendency of the index changes, especially affecting the extra-regional phenomenon. Inhabitants should avoid this expense getting thinner. Thus, we predominantly expect a negative relation between the *PHARM* and the extra-regional mobility of the patients. A third component affects the index and could be interesting in our research. The price of products is not the same everywhere, even if markets are basically the same all over Italy. The waste of money could be related to the simple wastage of resources or even to a bad contractual activity of the buyer. Thus, even *PHARM* measures the inefficient allocation of resources due to managerial incompetence. Hence, even if we stated earlier that the higher the *PHARM,* the better, we might find an inefficiency deterrent component in it that averts this kind of undesirable expenditure. A positive relation with the dependent variable is also possible, especially in migration within the region, due to the constancy of pharmaceutical spending policy within Apulia.

The last measure of costs we use is a linear combination of the others plus all residual costs of production (*TOTPROD* = item #B99999). That is the most synthetic index we could use to measure (in)efficiency in producing health care services. The index follows a constant growing path over the years. Variability among the groups diminishes over time. The trend in the BT HCA clearly shows the adaptation of the index to the average of other HCAs at the moment of the merger (2005–2006). Due to the comprehensive managerial significance of the total costs of production, it is basically addressed in literature as a measure of inefficiency due to relevant wastage [22].

### Econometric model

In order to investigate how the (in)efficiency in HCAs is related to the independent variables selected and explained above, we use infra-region and extra-region migration indexes in parallel (*ERM* and *IRM,* respectively) as proxies for (in)efficiency and test the following regressions under the pooled OLS Model:

(1a) $$\begin{array}{l} ERM_{i}=\beta_{0}+\beta_{1}OBTB_{i}+\beta_{2}DummyUNDER_{i}\\ \;+\beta_{3}DummyOVER_{i}+\beta_{4}OBPOP_{i}\\ \;+\beta_{5}MEDPERS_{i}+\beta_{6}OTHPERS_{i}\\ \;+\beta_{7}PHARM_{i}+\beta_{8}TOTPROD_{i}+u_{i} \end{array}$$

(1b) $$\begin{array}{l} IRM_{i}=\beta_{0}+\beta_{l}OBTB_{i}+\beta_{2}DummyUNDER_{i}\\ \;+\beta_{3}DummyOVER_{i}+\beta_{4}OBPOP_{i}\\ \;+\beta_{5}MEDPERS_{i}+\beta_{6}OTHPERS_{i}\\ \;+\beta_{7}PHARM_{i}+\beta_{8}TOTPROD_{i}+u_{i} \end{array}$$

Where, *OBTB* is the number of occupied beds to the number of available beds for each HCA; *DummyUNDER* is 1 if *OBTB* < 0.65, 0 otherwise; *DummyOVER* is 1 if *OBTB* > 0.89, 0 otherwise; *OBPOP* is the number of occupied beds to the resident population, *MEDPERS* are the costs of the medical personnel scaled by the resident population; *OTHPERS* are the costs of professional, technical, and administrative human resources scaled by population; *PHARM* are the costs for surgery and medical materials and pharmaceuticals to the population; and *TOTPROD* are total operating costs to the population. The meaning of the variables is better described in par. 3.2 and *i* varies across time (2001–2009) and space (HCAs of BA, BR, BT, FG, LE, TA). Data are run using Gretl and R statistical programs.

The unreported White test shows the need of robustness for hetheroskedasticity errors in both regressions. Thus, we apply the pooled OLS HAC model to the regressions and undertake the Hausmann panel diagnostic test. On both regressions (1a) and (1b), the adequacy of a Fixed Effects Panel Model compared to the Pooled OLS Model is noticed, being statistically significant to the time-demeaned effect of the intercepts. The successive Breusch-Pagan test suggests the Fixed Effects Panel Model is more adequate than the alternative Pooled OLS Model and Random Effects Panel Model.

(2a) $$\begin{array}{l} ERM_{\mathrm{it}}=\beta_{1}OBTB_{\mathrm{it}}+\beta_{2}DummyUNDER_{\mathrm{it}}\\ \;+\beta_{3}DummyOVER_{\mathrm{it}}+\beta_{4}OBPOP_{\mathrm{it}}\\ \;+\beta_{5}MEDPERS_{\mathrm{it}}+\beta_{6}OTHPERS_{\mathrm{it}}\\ \;+\beta_{7}PHARM_{\mathrm{it}}+\beta_{8}TOTPROD_{\mathrm{it}}+\alpha_{i}+u_{\mathrm{it}} \end{array}$$

(2b) $$\begin{array}{l} IRM_{\mathrm{it}}=\beta_{1}OBTB_{\mathrm{it}}+\beta_{2}DummyUNDER_{\mathrm{it}}\\ \;+\beta_{3}DummyOVER_{\mathrm{it}}+\beta_{4}OBPOP_{\mathrm{it}}\\ \;+\beta_{5}MEDPERS_{\mathrm{it}}+\beta_{6}OTHPERS_{\mathrm{it}}\\ \;+\beta_{7}PHARM_{\mathrm{it}}+\beta_{8}TOTPROD_{\mathrm{it}}+\alpha_{i}+u_{\mathrm{it}} \end{array}$$

where *t = 2001,…,2009* and *i = BA, BR, BT, FG, LE, TA*, and variables assume the meaning stated above.

We test errors for hetheroskedasticity in groups through the Wald test and find the variance of the errors being inconstant between groups [(2a): *χ*^*2*^*-stat* = 168.88 *p-value* 0.00; (2b): *χ*^*2*^*-stat* = 149.7 *p-value* 0.00].

Then, we adopt the model with robustness to hetheroskedasticity errors and find normally distributed idiosyncratic errors [*χ*^*2*^*-stat* = 0.898 with *p-value* = 0.63 in (2a) and *χ*^*2*^*-stat* = 2.44 with *p-value* = 0.63 in (2b)].

The model is quite consistent in both cases, with Adjusted-R^2^ = 0.73 and Akaike Criterion = −501.3 in (2a) and Adjusted-R^2^ = 0.97 and Akaike Criterion = −460.2 in (2b).

Coefficients for the regressions are in Table 5 and Table 6.

**Table 5:** **Fixed effects panel model (HAC)**$\begin{array}{l} ERM_{\mathrm{it}}=\beta_{1}OBTB_{\mathrm{it}}+\beta_{2}DummyUNDER_{\mathrm{it}}+\beta_{3}\text{DummyOVE}R_{\mathrm{it}}+\beta_{4}OBPOP_{\mathrm{it}}+\beta_{6}NOTHPERS_{\mathrm{it}}+\\ \beta_{7}PHARM_{\mathrm{it}}+\beta_{8}TOTPROD_{\mathrm{it}}+\alpha_{i}+u_{\mathrm{it}} \end{array}$

***Variable***	***Predicted sign***	***coeff.***	***p-value***
***Const***		0.0019	0.20
***OBTB***	+/−	0.0137	0.00
***DummyOVER***	+/−	−0.0031	0.00
***DummyUNDER***	+	0.0024	0.00
***OBPOP***	?	−0.0214	0.00
***MEDPERS***	-	0.0000	0.02
***OTHPERS***	+	0.0000	0.00
***PHARM***	-	−0.0000	0.12
***TOTPROD***	+	−0.0000	0.21
***Adj-R***^***2***^ 0.73		**Schwarz Cr.(BIC)** = −473	**Akaike Cr.(AIC)** = −501

**Table 6:** **Fixed effects panel model (HAC)**$\begin{array}{l} IRM_{\mathrm{it}}=\beta_{1}OBTB_{\mathrm{it}}+\beta_{2}DummyUNDER_{\mathrm{it}}+\beta_{3}DummyOVER_{\mathrm{it}}+\beta_{4}OBPOP_{\mathrm{it}}+\beta_{5}OTHPERS_{\mathrm{it}}+\beta_{6}NOTHPERS_{\mathrm{it}}+\\ \beta_{7}PHARM_{\mathrm{it}}+\beta_{8}TOTPROD_{\mathrm{it}}+\alpha_{i}+u_{\mathrm{it}} \end{array}$

***Variable***	***Predicted sign***	***coeff.***	***p-value***
***const***		−0.0189	0.09
***OBTB***	+/−	0.0148	0.02
***DummyOVER***	+/−	−0.0052	0.01
***DummyUNDER***	+	0.0060	0.00
***OBPOP***	?	0.0042	0.40
***MEDPERS***	-	−0.0001	0.00
***OTHPERS***	+	0.0001	0.42
***PHARM***	+	0.0000	0.00
***TOTPROD***	+	0.0000	0.01
***Adj-R***^***2***^ 0.97		**Schwarz Cr.(BIC)** = −432	**Akaike Cr.(AIC)** = −460

## Results

The Fixed Effects Panel Model we used shows a strong relationship between most of the independent variables we chose and the dependent ones. Here, we elaborate on the statistically significant results of the regressions, and discuss their economic usefulness.

Extra-regional migration (*ERM*) seems negatively influenced by the hospitalization index (*OBPOP*) with a coefficient of −0.02 (*p-value* = 0.00). Thus, while enhancing the number of hospitalized population, the extra-regional migration phenomenon decreases. Infrastructures are basically adequate to the populations’ needs. The issue is better investigated in the analysis of *OBTB*, joined with the analysis of *DummyUNDER* and *DummyOVER*. *OBTB* is in a positive relation with the extra-regional out-migration, with a coefficient equal to 0.013 (*p-value* = 0.00); an over-occupation of beds appears to produce a higher total migration, suggesting that overcrowding of hospital public structures might invite patients to go away. We check the evaluation of extreme cases of over-utilization with *DummyOVER*, which is negatively related to *ERM* (*coeff.* = −0.003, *p-value* = 0.00), consequently probably excluding the inadequacy of infrastructures as one probable reason of inefficiency. Intuitively, the more crowded the hospital, the greater might be its appeal to patients. The *DummyUNDER*, on the other hand, is positively related with out-migration (*coeff.* = 0.002, *p-value* = 0.00) as deserted hospitals seem to be a further cause of out-migration because patients do not trust those centers, probably due to prior bad personal or indirect experiences.

An interesting relationship occurs between cost regressors and the regressands. In the *ERM* regression, both kinds of labor costs (physician and non-physician) are positively related with the passive mobility phenomenon, measuring for *MEDPERS* a *coeff. =* 0.00005 (*p-value =* 0.02) and for *OTHPERS* a *coeff. =* 0.00018 (*p-value =* 0.00). Hence, spending for personnel does not make the HCA more competitive and patients cross the borders of the region notwithstanding the monetary efforts of their resident HCA in providing services. In particular, we expected a positive sign for *OTHPERS*, considering the administrative and general costs to be a probable waste. We did not expect a positive sign in *MEDPERS*. By the way, omitted variables probably affect the results of our analysis because we cannot individuate those cases that really need to pass over the borderline due to the specific disease they suffer and due to the circumstance that those services are not provided within Apulia. *PHARM* and *TOTPROD* are not statistically significant in the explanation of *ERM*. Thus, raising the expenditure is generally not appreciated by the population involved in the extra-regional migration.

In the explanation of *IRM*, *OBPOP* is not statistically significant, while the results of the analysis of *OBTB* (*coeff. =* 0.0148, *p-value* = 0.02), *DummyOVER* (*coeff. = −*0.0052, *p-value* = 0.01) and *DummyUNDER* (*coeff. =* 0.0060, *p-value* = 0.00) are consistent with the findings of the extra-regional observation. Thus, an overcrowded medical center seems to attract more patients than a deserted one, which is what we sensibly foresaw.

On the one hand, in the *IRM* regression, the expenditure on salaries for physician personnel diminishes the infra-regional migration phenomenon with a coefficient of −0.00012 (*p-value* = 0.00). Then, in the infra-regional migration, patients appreciate the expenditure on physician human resources, probably procuring a higher quality of health care services, as predicted.

On the other hand, the expenditure in medical and pharmaceutical provisions (*PHARM*) and the total cost of production (*TOTPROD*) are positively related to the migration index (*coeff.* = 0.0000 *p-value* = 0.00 and *coeff.* = 0.0000 *p-value* = 0.01, respectively); the more the HCAs spend, the less the patients trust the quality of their services. Hence, patients generally seem to consider every kind of expenditure in the local HCA as a waste of money and have different reasons to choose to leave their HCA. The Fixed Effects Panel Model for the regression picks up different constant terms for each cross-section, demeaning them differently by groups; the remaining motivations are contained in the *α*_*i*_ term and probably differ between cross-sections.

The difference in the evaluation of *PHARM* between patients migrating out of the region and within the regional borders (even if not statistically meaningful) could be due to the quite common pharmaceutical expense policy within the region. Thus, differences in expenditure within Apulia is probably due to waste. While choosing a different region can be due to different reimbursement policies, patients might evaluate a higher expense of their resident HCA positively when they have to decide to cross the regional borderlines. On the other side, they probably do not appreciate wastage when they choose between the different HCAs operating within the region.

Finally, we can state that some managerial inefficiency can be found in the overall production system, suggesting the possibility of a worrying waste of productivity in the procurement of materials or in their utilization. Henceforth, most migration is explained by infra-regional moving and the efficiency in spending can be a deterrent to the exodus. Nevertheless, patients crossing the regional limits might take their decision irrespective of the HCAs’ efforts in spending on personnel’s salaries to offer better services. By enhancing the costs, patients simply seem to be driven away.

### Robustness of the results

In order to assess the consistency of the reported results with the hypotheses, we conduct some additional tests to determine if any of our assumptions is likely to be false.

We conduct the sensitivity analysis in two ways.

First, we undertake a traditional backward elimination process [34]: 339–342] on the independent variables adopted in the model, dropping the controls first and the explanatory variables next (Table 7). The results of our analysis are basically unmodified when we drop, one by one, the independent variables. The predictive signs are basically confirmed at high significance levels and the Adjusted-R^2^ and Bayesian criteria keep consistent, notwithstanding the dropped variables.

**Table 7:** 1 variable backward sensitivity analysis

***ERM***	***Dropped variables***									
	***Original model (2a)***	***OBTB***	***DummyOVER***	***DummyUNDER***	***OBPOP***	***MEDPERS***	***OTHPERS***	***PHARM***	***TOTPROD***	
***OBTB***	+		-	0.01 (0.07)	0.00 (0.12)	0.00 (0.06)	0.01 (0.00)	0.01 (0.00)	0.01(0.00)	0.01 (0.00)
***DummyOVER***	-		−0.00 (0.03)	-	−0.00 (0.00)	−0.00 (0.01)	−0.00 (0.00)	−0.00 (0.00)	−0.00 (0.00)	−0.00 (0.00)
***DummyUNDER***	+		−0.00 (0.45)	0.00 (0.02)	-	0.00 (0.11)	0.00 (0.00)	0.00 (0.00)	0.00 (0.00)	0.00 (0.00)
***OBPOP***	-		−0.02 (0.00)	−0.02 (0.00)	−0.02 (0.00)	-	−0.01 (0.00)	−0.02 (0.00)	−0.02 (0.00)	−0.00 (0.00)
***MEDPERS***	+		0.00 (0.04)	0.00 (0.01)	0.00 (0.06)	−0.00 (0.59)	-	0.00 (0.04)	−0.00 (0.00)	0.00 (0.01)
***OTHPERS***	+		0.00 (0.00)	0.00 (0.00)	0.00 (0.00)	0.00 (0.04)	0.00 (0.00)	-	0.00 (0.00)	0.00 (0.00)
***PHARM***	-		−0.00 (0.19)	−0.00 (0.09)	−0.00 (0.08)	−0.00 (0.35)	−0.00 (0.99)	−0.00 (0.77)	-	0.00 (0.39)
***TOTPROD***	-		−0.00 (0.10)	−0.00 (0.19)	−0.00 (0.23)	0.00 (0.32)	0.00 (0.03)	0.00 (0.84)	−0.00 (0.15)	-
***Adj-R***^***2***^	0.73	0.67	0.72	0.72	0.58	0.69	0.71	0.74	0.73	
***BIC***	−473	−465	−472	−474	−451	−469	−471	−476	−476	
***AIC***	−501	−491	−498	−500	−477	−495	−497	−502	−502	
***IRM***	***Dropped variables***									
	***Original model (2b)***		***OBTB***	***DummyOVER***	***DummyUNDER***	***OBPOP***	***MEDPERS***	***OTHPERS***	***PHARM***	***TOTPROD***
***OBTB***	+		-	0.00 (0.26)	0.00 (0.96)	0.02 (0.02)	0.02 (0.00)	0.02 (0.02)	0.01 (0.06)	0.01 (0.00)
***DummyOVER***	-		−0.00 (0.11)	-	−0.00 (0.20)	−0.00 (0.00)	−0.00 (0.00)	−0.00 (0.02)	−0.00 (0.03)	−0.00 (0.00)
***DummyUNDER***	+		0.00 (0.12)	0.00 (0.03)	-	0.00 (0.00)	0.00 (0.00)	0.00 (0.02)	0.00 (0.00)	0.00 (0.00)
***OBPOP***	+		0.00 (0.18)	0.00 (0.31)	−0.00 (0.21)	-	−0.01 (0.02)	0.00 (0.36)	0.00 (0.35)	−0.00 (0.43)
***MEDPERS***	-		−0.00 (0.00)	−0.00 (0.00)	−0.00 (0.00)	−0.00 (0.00)	-	−0.00 (0.00)	−0.00 (0.00)	−0.00 (0.95)
***OTHPERS***	+		0.00 (0.42)	0.00 (0.59)	0.00 (0.00)	0.00 (0.39)	0.00 (0.28)	-	0.00 (0.26)	0.00 (0.44)
***PHARM***	+		0.00 (0.01)	0.00 (0.04)	0.00 (0.04)	0.00 (0.01)	0.00 (0.23)	0.00 (0.00)	-	0.00 (0.09)
***TOTPROD***	+		0.00 (0.00)	0.00 (0.00)	0.00 (0.00)	0.00 (0.00)	0.00 (0.03)	0.00 (0.00)	0.00 (0.00)	-
***Adj-R***^***2***^	0.97	0.97	0.97	0.97	0.98	0.97	0.97	0.97	0.96	
***BIC***	−432	−429	−430	−427	−436	−418	−434	−430	−431	
***AIC***	−460	−455	−456	−453	−462	−444	−460	−456	−439	

° In brackets: two-tails *p-values*.

When we drop two or more explanatory variables, the model starts losing significance.

In a second step, we undertake a different analysis to test the sensitivity of our model on a different sample. The main problem of robustness of our results depends on the low number of HCAs involved in the analysis (N = 6). Unfortunately, the data we used are not publicly released. We need to evaluate the firm-level of health care, avoiding the problem of comparing single hospitals and medical centers, which are extremely heterogeneous, differently sized, and generally specialized in treatments for particular diseases. Thus, we chose the “per-HCAs” level of disaggregation, in order to be able to give some critical remark from a managerial point of view. Those data (and the permission to elaborate and publish them) should be kindly conceded by regional or national health care authorities. We only obtained data and permissions from the Apulia Regional Authority. Thus, summing up the data of the (unobserved) HCAs, we get the (observed) regional heath care (RHC) analysis. The new sample size is 21, including all 20 Italian regions, 1 of which is separated into its 2 autonomous provinces.

Thus, the second block of robustness checks examines the sensitivity of our extra-regional migration analysis to:

i) sample selection

ii) proxy measurement choice of the dependent variable

iii) proxy measurement choice of the independent control variables

iv) functional form adopted in the regression

Shortly, we would test a different model specification on a different sample, with the aim of finding similar results in order to confirm our assumptions.

Regrettably, we have no possibility to change the four elements above step by step, due to a lack of the necessary data. Thus, we try a different analysis, changing all the elements at once.

*Sub i)*, we examine the sensitivity of our results in equation (2a) to a different sample, aggregating HCAs at a regional level. In the meantime, in *sub ii)*, we use the *MIGPOP* index measured in our analysis to consider the number of patients migrating in and out, and the length of the treatment. It does not consider difficulties in care-giving. Thus, in this sort of robustness test, we adopt a proxy of (in)efficiency in health care based on three different elements (the number of patients *times* the length of treatment *times* the difficulty of treatment). *Sub iii)*, we drop the *OBTB, DummyOVER,* and *DummyUNDER* because those data are not available. Finally, in *sub iv)*, we use a different model to fit the data, changing Fixed Effects Panel Model to Random Effects Panel Model. The Random Effects is not applicable to the Apulia sample due to insufficient degrees of freedom, but is more suitable for the Italian sample.

Basically, the results of our analysis on the total Italian sample, with slightly different variables, remain similar to those reported in Table 5 and the predicted signs are confirmed in statistically relevant variables (Table 8).

**Table 8:** **Random effects panel model (GLS) with Italian regional sample***ERM*_*it*_ = *β*_1_*OBPOP*_*it*_ + *β*_2_*MEDPERS*_*it*_ + *β*_3_*OTHPERS*_*it*_ + *β*_4_*PHARM*_*it*_ + *β*_5_*TOTPROD*_*it*_ + *α*_*i*_ + *u*_*it*_

***Variable***	***Sign in original model (2a)***	***coeff.***	***p-value***
***Const***		−7.9653	0.19
***OBPOP***	+	0.0489	0.04
***MEDPERS***	-	6.7046	0.46
***OTHPERS***	-	−69.663	0.07
***PHARM***	+	11.001	0.00
***TOTPROD***	+	−1.8516	0.47
		**Schwarz Cr.(BIC)** = 1480	**Akaike Cr.(AIC)** = 1461

*** >0.99% significance level; ** > 0.95% significance level; * >0.90 significance level.

The robustness of the findings helps to indentify some critical assumptions and to assess the validity of the model not only in Apulia regional borders, but all over the Italian health care system, involving both the areas affected by passive (like Apulia) and active migration phenomena.

## Conclusion

This paper investigates the behavior of patients in relation with expenditure in public health care.

The decision of patients to leave their HCA and move far from home in order to get a health treatment is not a casual decision. The fact that some areas in Italy (especially in southern Italy) regularly experience severe passive performances in migration indexes is extremely meaningful. We found regular and consolidated relations between some cost and activity indicators and the passive mobility phenomenon, adding some empirical evidence to the literature. To the best of our knowledge, no prior research tests the sensibility of patients to the way the HCA allocates its resources. Thus, we argument and predict correlations between variables and find evidence of a certain relevance of the issue.

Spending in the proper way on health care could bring about an enhancement of performances. Instead, wasting resources seems to be immediately perceived by the patient and probably leads him to an adverse behavior.

Our first hypothesis seems to be confirmed in the regression analysis. Different ways of spending (i.e. different managerial choices of resources’ allocation) appear to produce an effect on citizens. Patients seem to evaluate the spending policies adopted by their resident HCAs, and, “voting by their feet”, convey the impression of out-migrating when the HCA spends improperly, wasting money in costs that are not critical to the quality of the health care service provided. Even our second hypothesis seems to be confirmed by results. The “voting by feet” choice seems more sensitive to various managerial patterns of spending when the distance from the alternate HCA is shorter. Thus, it is feasible that the infra-regional mobility is strongly and differently influenced by various spending choices. On the other side, the extra-regional mobility apparently is influenced by wasting in general and might lead the patient to out-migrate when the total operating costs arise. Different possible ways of spending are less relevant. Thus, the spatial element influences the “vote by feet” and we need to deflate the observation of the phenomenon of mobility in order to mitigate the measure of distances.

Further investigation could be interesting. First, the enlargement of the sample, broadening the perimeter of the analysis even to the active-migration areas, could ameliorate our thesis and consolidate our findings. The unusual robustness test we adopt using regional disaggregated data predicts the consistency of our findings at a national level. Nevertheless, the analysis on the specific sample of Apulia is a cogent addition to literature, as few studies of efficiency in health care have been undertaken in Italy before and only one in Apulia.

Second, a more capillary analysis might add other independent variables, testing other relevant components of the productivity in health care, like investments in medical devices, length of waiting lists, and surveys on the satisfaction of inpatients and outpatients. Unfortunately, data on those aspects are neither easy to collect nor provided by the regional or national authorities.

## Endnote

^a^Other autonomous public hospitals, clinics, and medical centers operating on the same territory of the target HCA are not included in this analysis.

## Competing interests

The authors declare that they have no competing interests.

## Authors’ contributions

EM did substantial contributions to the project conception and design, participated in the data acquisition and in the analysis and interpretation of data, prepared the draft of the article and approved the final version. ED’A did substantial contributions to conception and design, revised the manuscript critically for important intellectual content and approved the final version. Both authors read and approved the final manuscript. Authors gratefully acknowledge for helpful comments Prof. Roberto Dell’Anno, two anonymous referees, participants at EURAM 2012 Conference in Rotterdam Erasmus University and participants at Public Sector Conference 2012 in Milano Bocconi University. Furthermore, the paper has benefitted from the kind provision of data by the Apulia Regional Authority and the Svim Service Srl.
